# Supplementary material for: Database of virtual objects to be used in psychological research
Source: PLoS One. 2020 Sep 4;15(9):e0238041. doi: 10.1371/journal.pone.0238041 (PMC7473576; doi:10.1371/journal.pone.0238041)
Supplement: Attachment — Submitted filename: review 20200607.pdf [file pone.0238041.s003.pdf]

The purpose of this research is clear, the design and procedure are straight forward, and the methodology is consistent with some other researches in this area. These are the strength of this paper.

In terms of significance, virtual reality does play a more and more important role in psychological experiments nowadays. Therefore, building good databases, especially the first one with 3-D objects, is truly important work.

However, there are several questions I see in this paper. The first concern is about the size of the stimuli. As a “database”, 121 are not quite enough, to begin with. By further examining the objects, it seems 33 objects have more than one version. For example, there are five chairs in the database. Take this into consideration, there are only 82 objects/concepts included. A database with such a small size would limit its practical implication. (e.g., In the references of this paper, [8] Snodgrass & Vanderwart had 260 pictures; [9] Bates et. al. had more than 700 pictures. And they are not even “databases”). I would suggest some rephrasing.

The second concern is about the categories in use. The 121 objects belong to furniture, electric device, and food. Why these three? Why only these three? Do they have enough power to represent a database of objects for common daily life?

Another question is about the participants. 12 non-US participants were removed because they were not “the target population”. I was a bit confused about the reason why using citizenship instead of language (English) proficiency as the criterion. Especially the authors did mention the 83 participants’ English is well enough to understand the procedure and questions.

This paper aimed to “create, standardize, and share the first set of objects” for psychological experiments. Does the “standardizing” refer only to the modal names? Does it have any psychometric sense in it?

In sum, the issue is important and the methodology is fine. However, the database is small, as well as there are some conceptual concerns to be clarified.
